# Economic Conditions in Nurse-Training

**Published:** 1916-07-08

**Authors:** 


### THE MATRONS' AND SISTERS' DEPARTMENT.

### ECONOMIC CONDITIONS IN NURSE-TRAINING.

For some years with increasing persistence it has been driven home to the authorities of the great voluntary hospitals to which are attached large training-schools for nurses that the time is approaching, if it has not already arrived, when it will be essential to cry a halt so far as the number of probationers taken into the schools is concerned. The cost entailed upon the voluntary hospitals by the training of probationers under modern conditions has steadily increased until it has arrived at a point where any further extension must be prevented, or the cost per occupied bed will become burdensomely excessive. The capital cost of providing a new up-to-date Nurses' Home as a part of each hospital establishment is another point which hospital administrators have to keep constantly before them. Further, the number of probationers which a large hospital is justified in attempting to train must, in justice to the probationers and to the reputation of each school, be limited by the number of occupied beds and the facilities for training which each establishment can properly afford. All these matters are approaching a point where a crisis must arise.

The nature of the crisis will be readily understood when we point out that after the war there is little doubt that the demand for trained nurses, which has been increasing steadily of late years, will multiply, and may attain huge proportions. Those who will be responsible for the policy pursued by the College of Nursing will be called upon to go very closely into the problem just indicated, and it will be no small part of their responsibility to help to find a practical solution to many of the difficulties presented. At the end of the war, up and down the country there will be a large number of hospital buildings which had no existence at its commencement. When the demand for hospital accommodation for sick and wounded warriors ceases, many of these buildings should become available for the treatment of civilians. We have long advocated the conversion of the best type of Poor-Law infirmaries, many of which have been hygienically and in the hospital sense enlarged and improved to fit them for the reception of the sick and wounded from the war. Other buildings of the same class will be found in the neighbourhood of most of our great cities and in various rural districts. There will therefore be many hospital beds and much accommodation which might be made available readily for the use of the civil population. The growth in surgery has brought an ever-increasing demand for hospital care amongst all classes of the civil population, and there is a widespread feeling that facilities should be granted them to obtain hospital care and treatment on payment. Here, as it seems to us, are the means to solve the economic problem of further centres for the training of probationers and for the provision of the much-needed hospital care and accommodation for all classes of the people.

The discussion instituted by Miss Pearse at the conference last week of the National Union of Trained Nurses on the Economic Conditions of the Nursing Profession and the points which were urged by herself and Mrs. Bedford Fenwick are much to the point. The former insisted that the present scale of payments for trained nurses was inadequate. This objection would lose much of its point if the increases in the pay of hospital sisters and charge nurses which have prevailed during war-time were to be made permanent. The position of certificated trained nurses engaged in private practice must be materially benefited when State recognition is obtained through the passing of an agreed Registration Bill. That Bill should contain a clause empowering the General Nursing Council to move the County Councils to inspect and close so-called nursing homes which supply the public with so-called nurses who are in no sense properly trained, or who are inadequately trained, some of whose characters and history may require close investigation. What character in any right meaning of the word can the conductors of such establishments, and the women who have not been properly trained and are not competent as fully trained nurses, claim to possess? Mrs. Bedford Fenwick, with justice, insisted that candidates for training must understand that the higher and better the training the more essential is it that they should recognise its cost. This cost made full training an adequate return for the services rendered during its course. Economically it is not a practical proposal to suggest that probationers should receive free instruction and training and also be paid for their services. Miss Pearse made a good point, too, in insisting that the admission of paying pupils meant an increase in the number of ward-maids and an increased cost to each school. We hope that the matrons of the larger hospitals may take an opportunity, in conjunction with their authorities, to hold conferences of nurses and to explain to them these economic problems, with others which we have no space to enter into here.

